# Cellular Mechanisms Accounting for the Refractoriness of Colorectal Carcinoma to Pharmacological Treatment

**DOI:** 10.3390/cancers12092605

**Published:** 2020-09-11

**Authors:** Jose J.G. Marin, Rocio I.R. Macias, Maria J. Monte, Elisa Herraez, Ana Peleteiro-Vigil, Beatriz Sanchez de Blas, Paula Sanchon-Sanchez, Alvaro G. Temprano, Ricardo A. Espinosa-Escudero, Elisa Lozano, Oscar Briz, Marta R. Romero

**Affiliations:** 1 Experimental Hepatology and Drug Targeting (HEVEFARM), University of Salamanca, Salamanca Biomedical Research Institute (IBSAL), 37007 Salamanca, Spain; rociorm@usal.es (R.I.R.M.); mjmonte@usal.es (M.J.M.); elisah@usal.es (E.H.); anapeleteiro@usal.es (A.P.-V.); beatrizsanchezbla@usal.es (B.S.d.B.); pausanchons@usal.es (P.S.-S.); alvarogacho@usal.es (A.G.T.); raespinosa@usal.es (R.A.E.-E.); elisa_biologia@usal.es (E.L.); obriz@usal.es (O.B.); 2 Center for the Study of Liver and Gastrointestinal Diseases (CIBERehd), Carlos III National Institute of Health, 28029 Madrid, Spain

**Keywords:** apoptosis, cancer stem cell, colon cancer, DNA repair, drug transport, epithelial–mesenchymal transition, genetic variants, metabolism, multidrug resistance, tumor environment

## Abstract

**Simple Summary:**

Colorectal cancer (CRC) causes a high number (more than 800,000) of deaths worldwide each year. Better methods for early diagnosis and the development of strategies to enhance the efficacy of the therapeutic approaches used to complement or substitute surgical removal of the tumor are urgently needed. Currently available pharmacological armamentarium provides very moderate benefits to patients due to the high resistance of tumor cells to respond to anticancer drugs. The present review summarizes and classifies into seven groups the cellular and molecular mechanisms of chemoresistance (MOC) accounting for the failure of CRC response to the pharmacological treatment.

**Abstract:**

The unsatisfactory response of colorectal cancer (CRC) to pharmacological treatment contributes to the substantial global health burden caused by this disease. Over the last few decades, CRC has become the cause of more than 800,000 deaths per year. The reason is a combination of two factors: (i) the late cancer detection, which is being partially solved by the implementation of mass screening of adults over age 50, permitting earlier diagnosis and treatment; (ii) the inadequate response of advanced unresectable tumors (i.e., stages III and IV) to pharmacological therapy. The latter is due to the existence of complex mechanisms of chemoresistance (MOCs) that interact and synergize with each other, rendering CRC cells strongly refractory to the available pharmacological regimens based on conventional chemotherapy, such as pyrimidine analogs (5-fluorouracil, capecitabine, trifluridine, and tipiracil), oxaliplatin, and irinotecan, as well as drugs targeted toward tyrosine kinase receptors (regorafenib, aflibercept, bevacizumab, cetuximab, panitumumab, and ramucirumab), and, more recently, immune checkpoint inhibitors (nivolumab, ipilimumab, and pembrolizumab). In the present review, we have inventoried the genes involved in the lack of CRC response to pharmacological treatment, classifying them into seven groups (from MOC-1 to MOC-7) according to functional criteria to identify cancer cell weaknesses. This classification will be useful to pave the way for developing sensitizing tools consisting of (i) new agents to be co-administered with the active drug; (ii) pharmacological approaches, such as drug encapsulation (e.g., into labeled liposomes or exosomes); (iii) gene therapy interventions aimed at restoring the impaired function of some proteins (e.g., uptake transporters and tumor suppressors) or abolishing that of others (such as export pumps and oncogenes).

## 1. Introduction

Data collected in 2018 by the Global Cancer Observatory (GCO, https://gco.iarc.fr), supported by the International Agency for Research on Cancer (IARC) of the World Health Organization (WHO), revealed that colorectal cancer (CRC) constitutes a severe health burden, causing substantial mortality and morbidity worldwide. The CRC incidence in 2018 was 1,849,518 new cases worldwide. The 1-year prevalence was 1,356,151 patients, whereas the mortality was 880,792 individuals (55% males and 45% females). These data placed CRC in the fourth position regarding global cancer incidence, after breast, prostate, and lung cancer, but only the second (after breast cancer) regarding 1-year prevalence. Taking into account the ranking of most lethal cancers, CRC is located second after lung cancer. More detailed analysis of available data revealed a positive association between CRC morbidity/mortality and socioeconomic status [1], which is consistent with the impact of dietary and lifestyle risk factors on the etiology and pathogenesis of CRC [2], added to the substantial heritable component involved in the risk of developing this malignancy [3]. Patients with advanced CRC often present evident symptoms, but tumors at early stages of development and premalignant adenomatous polyps are commonly asymptomatic. This makes it difficult to detect them when curative options are feasible. 

**Figure 1:**
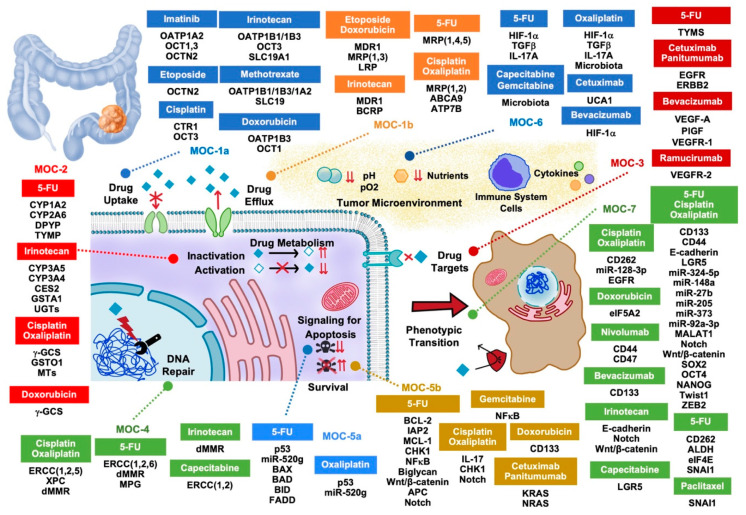
Proteins, non-coding RNAs, and signaling pathway regulators involved in the lack of response of colorectal adenocarcinoma to pharmacological treatment. MOC, mechanism of chemoresistance.

In the last few years, the implementation by many national health systems of mass screening of adults over age 50 has unraveled many of these cases, allowing treatment at an early stage, saving many lives. Although, chemoprevention is not currently the standard medical practice, the use of available prophylactic agents to reduce the risk of CRC in specifically selected populations offers a complementary strategy to colorectal surveillance [4]. The treatment of choice for CRC is surgery with laparoscopic resection of the affected segment [5]. Moreover, neoadjuvant radiotherapy is efficient in lowering local–regional recurrences [6]. Regarding the usefulness of available pharmacological tools, both conventional chemotherapy, mainly based on 5-fluorouracil (5-FU) and other pyrimidine analogs (e.g., capecitabine, trifluridine, and tipiracil), platinated agents (e.g., oxaliplatin), irinotecan, and drugs targeted toward tyrosine kinase receptors (e.g., regorafenib, aflibercept, bevacizumab, cetuximab, panitumumab, and ramucirumab) provide only scarce beneficial effects in some patients with advanced disease (i.e., stages III and IV). Therapies based on immune checkpoint inhibitors or anti-programmed cell death ligand 1 (PD-L1) drugs (e.g., nivolumab, ipilimumab, and pembrolizumab) have recently been approved by the FDA for use against CRC [7]. Unfortunately, the existence of strong mechanisms of chemoresistance (MOC) before starting the treatment (primary or innate chemoresistance) or developed in response to the pharmacological challenge (secondary or acquired chemoresistance) hampers the satisfactory outcome of CRC patients. This feature is also present in other tumors affecting the liver and digestive system, such as hepatocellular carcinoma [8], cholangiocarcinoma [9], hepatoblastoma [10], and adenocarcinomas of pancreas [11] and stomach [12]. In the present review, we have inventoried the genes involved in the lack of CRC response to pharmacological treatment based on their classification into seven groups (from MOC-1 to MOC-7) according to functional criteria (Figure 1).

## 2. Drug Uptake and Export (MOC-1)

Proteins involved in the transport of drugs and xenobiotics across the healthy intestinal epithelium may play a crucial role in the development of chemoresistance due to a decreased uptake (MOC-1a) or an increased efflux (MOC-1b) of anticancer agents (Table 1).

### 2.1. Drug Uptake Carriers (MOC-1a)

Several organic anion-transporting polypeptides (OATPs), belonging to the gene superfamily of solute carriers for organic anions (*SLCO*), expressed in the small intestine and colon, may play a role in the refractoriness to pharmacological treatment, as they participate in the uptake of a large variety of drugs involved in the treatment of CRC [11,13]. Polymorphisms in OATP1B1 (*SLCO1B1*), such as the OATP1B1*15 haplotype [11,14], both alone and accompanied by variants in other carriers, influence the response to methotrexate [15,16] and irinotecan in vitro [16,17], due to reduced transport activity; for the latter, this is the case in metastatic CRC patients, where it is used as a palliative treatment. Patients with the SLCO1B1*15 polymorphism showed higher systemic exposure and lower clearance of SN-38 [18,19]. 

Expression levels of OATP1B3 (*SLCO1B3*) in healthy colon and polyps of the large intestine are low [14,15,20], which is directly related to the sensitivity to methotrexate [21], irinotecan [22], and doxorubicin [23]. However, a form of alternative splicing of this carrier, lacking the first 28 amino acids, called cancer-type OATP1B3, is markedly expressed in colon cancer tissue [24,25], which could affect the sensitivity of tumor cells to OATP1B3 substrates and has been associated with poorer clinical response to irinotecan therapy in CRC patients [26].

The expression of OATP1A2 (*SLCO1A2*), involved in the uptake of imatinib, but not other tyrosine kinase inhibitors (TKIs) [27], is markedly reduced in tumors as compared to healthy colon tissue [14,15,20]. Among the OATP1A2 polymorphisms studied, four showed altered transport of methotrexate in vitro. The common I13T OATP1A2 SNP showed enhanced methotrexate uptake, whereas R168C, E172D, and N278del variants showed significantly decreased methotrexate uptake. However, as suggested by in vitro experiments, neither imatinib nor methotrexate response has been associated with loss-of-function OATP1A2 variants [16,28]. 

Members of the SLC22 family, belonging to the groups of organic anion (OAT) and cation (OCT) transporters, can participate in the uptake of methotrexate, imatinib, and some platinum derivatives, such as oxaliplatin, but not cisplatin and carboplatin. A reduction in OCT expression has been related to the loss of sensitivity to these drugs [29,30,31]. However, the clinical significance of these carriers in CRC chemoresistance is not well established yet. The expression and uptake efficacy of OCT1 (organic cation transporter 1, *SLC22A1*) is reduced in polyps and CRC [32,33]. These changes have been associated with reduced sensitivity and cellular response to imatinib in other tumors [34,35]. Moreover, OCT1 may be involved in the uptake of doxorubicin [11,36], although its relevance in drug resistance in CRC must be elucidated. Several studies have suggested that OCT3 levels (*SLC22A3*) are reduced in intestinal tumors [32,33], which may be involved in the lack of response found in clinical practice in the treatment of other digestive tumors using irinotecan, cisplatin, and several TKIs, such as imatinib [37,38]. On the contrary, high expression of OCT3 could be involved in the low response to FOLFOX regimen (folinic acid, 5-FU, and oxaliplatin) [39], suggesting that other mechanisms different from drug uptake through OCT3 must account for this resistance.

OCTN1 (*SLC22A4*) participates in the uptake of doxorubicin, mitoxantrone, and oxaliplatin [13,40]. However, its expression is sharply reduced in intestinal tumors [14,32]. Besides, the *SLC22A4* variant c.1672C > T (rs1050152) is associated with ulcerative colitis, sporadic CRC, and increased risk for CRC development. This variant has been suggested as a useful biomarker to predict malignant progression [41]. Polymorphisms rs2631367 and rs2631372, described in the *SLC22A5* gene encoding OCTN2, another member of the SLC22 family involved in etoposide [42] and imatinib [43] transport, have been related to the prognosis of some gastrointestinal tumors treated with imatinib [44].

The human copper transporter 1 (CTR1, *SLC31A1*) is downregulated in CRC cells after exposure to cisplatin [25]. This transporter is determinant for cellular accumulation and toxicity of cisplatin and carboplatin [45,46]. However, results regarding the relationship between CTR1 and sensitivity to oxaliplatin are controversial, since another type of resistance mechanism independent of CTR1 could be involved in refractoriness to oxaliplatin [45,46].

Carriers of the SLC19 family are involved in methotrexate uptake in vitro [30]. Accordingly, their decreased expression has been associated with enhanced resistance to this drug in CRC tumors [30]. In addition, the *SLC19A1* variant rs1051266 combined with the *SLCO1B1* variant rs2306283 seem to be related to a higher response rate to irinotecan in metastatic CRC patients [18].

In summary, carriers OATP1B1, OATP1B3, OCT1, and OCT3 are involved in the uptake of antitumoral drugs with clinical relevance in CRC treatment.

### 2.2. Drug Export Pumps (MOC-1b)

Members of the ATP-binding cassette (ABC) superfamily of proteins are involved in resistance to pharmacological treatment, leading to decreased intracellular drug concentrations and, hence, loss of therapeutic effects. Multidrug resistance protein 1 (P-glycoprotein or MDR1, gene symbol *ABCB1*) is highly expressed in healthy colorectal mucosa, polyps, and CRC cells [14], where it prevents the accumulation of a wide variety of drugs [47,48,49], thus contributing to the underlying mechanisms involved in chemoresistance [4,50,51]. Besides, the *ABCB1* polymorphisms c.3435C > T (rs1045642), c.2677G > T/A (rs2032582), and c.1236C > T (rs1128503) described in CRC patients have accounted for altered substrate affinity in vitro, including antitumor drugs. However, results verifying genotype correlations with allele and haplotype analysis are inconsistent, so further studies are needed [4,11,50,51,52]. 

Classical resistance modifying agents, such as verapamil, and emerging new TKI-based treatments sensitize CRC cells to typical substrates of ABC pumps (i.e., doxorubicin) [53]. Moreover, overexpression of MACC1 (metastasis-associated in colon cancer 1) in CRC, which is able to interact with the *ABCB1* promoter, increased its transcriptional activity in vitro, resulting in elevated MDR1 expression and thus enhanced resistance to conventional pharmacological therapies [54].

The expression of MDR-associated proteins (MRPs), such as MRP1 (*ABCC1*) and MRP3 (*ABCC3*), is, in general, reduced in cancerous tissues compared to healthy intestinal epithelia. This is not the case for MRP2 (*ABCC2*), whose mRNA levels are markedly increased in CRC tissue [55,56].

Despite the fact that most of them may be repressed in CRC tumors, it has been shown that, when expressed in vitro, these efflux pumps can confer CRC cell resistance to a wide variety of cytostatic agents, such as cisplatin (MRP2), doxorubicin (MRP1 and MRP3), and etoposide (MRP1 and MRP3). Although studies regarding 5-FU, irinotecan, and its active metabolite SN-38 may point to a possible role of these pumps in their response to chemotherapy in CRC patients, in vivo studies to support development of these proteins as targets for therapy are needed [30,56,57,58,59]. 

The ability of MRP1 to confer resistance to doxorubicin [61], 5-FU, and oxaliplatin [62] has been demonstrated in different CRC cell lines transfected with this carrier. However, its clinical relevance in CRC refractoriness to antitumor chemotherapy remains to be established. MRP2 is considered a crucial mechanism associated with resistance to cisplatin treatment in CRC [56]. Thus, a markedly increased expression of MRP2 appeared after incubating CRC cells with cisplatin [11,72]. However, a correlation between MRP2 expression levels and disease severity or prognosis has not been established [59,63]. MRP3 expression has been associated with doxorubicin and etoposide resistance in several gastrointestinal cell lines, including some derived from CRC, probably through a mechanism that involves the Wnt/β-catenin signaling pathway [63,73]. Pharmacological pressure upregulates this transporter, which can contribute to developing acquired drug resistance in CRC patients [63], similar to that described in other enterohepatic tumors, such as cholangiocarcinoma [74].

Polymorphism rs3742106 in the 3’-UTR region of *ABCC4* mRNA determines the sensitivity of CRC patients to 5-FU/capecitabine-based chemotherapy, individuals with the T/T genotype being more sensitive than those with G/G and G/T genotypes [64]. Moreover, MRP4 expression has previously been associated with resistance to camptothecins in vitro [75].

MRP5 (*ABCC5*), which is overexpressed in CRC, confers resistance to 5-FU in vitro through the active efflux of its monophosphate metabolites [76]. Celecoxib, a nonsteroidal anti-inflammatory drug (NSAID) used in combined treatments with irinotecan and 5-FU against CRC cancer, can upregulate both MRP4 and MRP5, reducing anticancer response to these drugs in vitro [77].

Polymorphisms c.34G > A (rs2231137) and c.421C > A (rs2231142) affecting the *ABCG2* gene, encoding the breast cancer resistance protein (BCRP), in combination with those found in methylenetetrahydrofolate reductase (MTHFR), may be useful to select oxaliplatin-based chemotherapy, such as FOLFOX and XELOX (oxaliplatin and capecitabine) versus FOLFIRI (folinic acid, 5-FU, and irinotecan), in patients with metastatic CRC [66]. This is due to BCRP upregulation, which has been shown to confer drug resistance against irinotecan and SN-38 and influences negatively due to a reduced response and progression-free survival (PFS) in CRC patients [51,67].

Due to the ability of P-type ATPases, ATP7A (Menkes’ protein), and ATP7B (Wilson’s protein) to export platinum derivatives, they have been suggested to play a role in the development of resistance to oxaliplatin [4,11]. High levels of *ATP7B* mRNA expression have been associated with poor outcome in CRC patients receiving oxaliplatin-based chemotherapy [68].

Several members of the ABCA family have been related to resistance in CRC due to their high expression levels. Thus, *ABCA9* polymorphisms were associated with reduced survival in CRC patients who received oxaliplatin-based chemotherapy [69], whereas ABCA13 overexpression may be associated with improved outcomes in CRC, due to an increment in the disease-free interval of patients treated by adjuvant chemotherapy which have ABCA13 upregulated in tumor tissues [59].

Although the lung resistance-related protein LRP (*MVP*) is not a pump, it has been associated with the reduction of intracellular levels of active agents by drug sequestration. In tumors and cells derived from CRC, LRP has been related to resistance to doxorubicin and etoposide [70,71] but not to 5-FU [78]. 

Taking into account all the above-mentioned clinical results, it could be concluded that MRP4, BCRP, ATP7B, and ABCA9 may play an important role in the development of chemoresistance in CRC patients.

## 3. Drug Metabolism (MOC-2)

Changes in phase I and phase II enzymes involved in drug metabolism by cancer cells can result in decreased pharmacological action, either by enhanced generation of inactive metabolites or diminished activation of prodrugs. These processes are classified as MOC-2 (Table 2). 

CYP450 enzymes are key players in the phase I-dependent metabolism of many compounds, which mediates the activation of numerous prodrugs and the inactivation of anticancer agents. Thus, the overexpression of CYP450 enzymes in tumor cells can reduce the efficacy of chemotherapeutic agents, such as irinotecan, that is inactivated by CYP3A4 [79] and CYP3A5 [80] in CRC cells, where their expression is significantly higher in non-responsive patients. Moreover, tumor cells develop a high capacity to inactivate antitumor drugs during treatment by increasing the expression of these enzymes. Thus, CYP1A2 and CYP2A6 were found significantly upregulated in 5-FU-resistant CRC cells, whereas 5-FU cytotoxicity was partially restored in vitro by co-treatment with a CYP450 inhibitor [81].

Carboxylesterases (CES), key enzymes in the intracellular activation of irinotecan, are consistently downregulated in CRC cells with enhanced resistance to this drug [82]. Several reports have positively correlated the degree of CES2 expression in tumor tissues with the efficacy of irinotecan-based therapy of CRC [83,84]. Moreover, many single nucleotide polymorphisms (SNPs) in the *CES2* gene that generate an inactive or less active protein have been described. Interestingly, some of them, such as c.-823C > G (rs11075646), located in the 5’-UTR region of *CES2* mRNA that enhances its transcription, are related to an increased response to capecitabine and a slow tumor progression in CRC patients [85]. Recently, in vitro studies associated *CES2* expression with CRC response to oxaliplatin [86]. Thus, experimental *CES2* downregulation in oxaliplatin-resistant CRC cells reversed their resistance, inhibited cell growth, and induced apoptosis by suppressing the AKT signaling pathway. Based on these results, *CES2* was proposed as a useful biomarker and therapeutic target to overcome oxaliplatin resistance in CRC [86].

As dihydropyrimidine dehydrogenase (*DPYP*) is involved in 5-FU catabolism, its elevated expression may lead to 5-FU resistance in CRC tumors [87]. In some patients, *DPYP* upregulation may be attributed to abnormal miR-494 downregulation, which results in an enhanced DPYP function and an accelerated rate of 5-FU inactivation [88]. Thus, in order to overcome tumor cell chemoresistance through increased 5-FU bioavailability, several strategies have been developed, aiming at reducing DPYP activity, such as its blockage with the pyrimidine analogue eniluracil in patients [89], or suppressing DPYD expression through the use in vitro of different synthetic transcription inhibitors (i.e., sphingosine-1-phosphate G-coupled receptor 2 or S1PR2 inhibitors) [90].

Other mechanisms affecting the response to 5-FU include the high expression of thymidine phosphorylase gene (*TYMP*). This is the first enzyme in the metabolic activation of 5-FU whose degree of expression has been directly related to the sensitivity to 5-FU in CRC patients [87]. It has been demonstrated that bevacizumab upregulates *TYMP*, thus enhancing the accumulation and cytotoxicity of 5-FU in CRC. Accordingly, use of bevacizumab synergistically with fluoropyrimidine-based chemotherapy to treat these tumors has been proposed [91].

Conjugation with glutathione, which is catalyzed by glutathione-S-transferases (GSTs), plays a crucial role in detoxifying endogenous and xenobiotic compounds. As a consequence of conjugation with glutathione, anticancer drugs are inactivated and become more water-soluble, which promotes their elimination as urine or bile [100]. GST overexpression and the existence of polymorphisms are found in several cancers and have been associated with chemoresistance. Thus, GSTA1 plays an essential role in irinotecan resistance in CRC cells [94]. In CRC patients treated with 5-FU and oxaliplatin, GSTP1*B polymorphism (c.313A > G, rs1695) has been associated with an increase in overall survival (OS) [101]. Moreover, in vitro studies have shown that an increase in GSTP1 expression may determine higher refractoriness to anthracyclines [92]. Moreover, higher availability of GSH due to a rise in the expression of γ-glutamylcysteine synthetase (γ-GCS), the key enzyme in GSH synthesis, has been related to doxorubicin [92] and cisplatin [93] resistance in CRC cell lines and patients, respectively.

Omega GSTs (GSTO1 and GSTO2) belong to an atypical cytosolic class of enzymes that, instead of glutathione conjugation, catalyze other thiol transfer reactions. GSTO1 overexpression has been reported in CRC patients [102], and in vitro studies relate this condition to cancer progression and development of drug resistance, particularly to platinum-containing compounds, by activation of survival pathways (AKT and ERK1/2) and inhibition of apoptotic mediators (JNK1) [95]. The GSTO1 inhibitor C1-27 can suppress cell growth and enhance cisplatin-induced cytotoxic effects in CRC cells and patient-derived xenografts [103].

SN-38 is inactivated in CRC cells by UDP-glucuronosyltransferases (UGTs)-mediated conjugation with glucuronic acid [97]. Thus, enhanced UGT-dependent conjugating activity, through UGT1A1 and UGT1A4, may contribute to CRC resistance to irinotecan [96,97]. Moreover, several polymorphisms of the *UGT1A1* gene have been associated with the clinical efficacy of irinotecan [104,105] and increased risk of CRC development [106].

Intracellular levels of metallothioneins (MTs) involved in apoptosis activation, DNA protection, and oxidative stress damping [107] have been associated with the development of resistance to chemotherapy in CRC. High levels of expression and functionality of MTs have been related to the reduced sensitivity of CRC cells to cisplatin [98]. Therefore, although there is some controversy regarding this concept, MT expression has been proposed as a marker of poor prognosis in CRC patients [99,108]. As such, available clinical data on SNPs and the expression of the genes encoding the proteins of the drug metabolism reveal valuable biomarkers that can lead to the customization of chemotherapeutic regimens and the selection of appropriate antitumor agents based on its chance of success depending on each tumor’s characteristics.

## 4. Changes in Drug Targets (MOC-3)

In the management of CRC patients, crucial mechanisms of resistance to chemotherapy, targeted therapy, and immunotherapy are due to alterations in drug targets (Table 3).

The enzyme thymidylate synthase (TYMS) plays an essential role in the de novo synthesis of DNA. Besides, it is the target of 5-FU and capecitabine metabolites, which inhibit TYMS as part of their mechanism of action. Thus, the determination of TYMS expression levels in CRC could be useful to select candidate patients for treatments with these fluoropyrimidines. Nevertheless, the results regarding this issue are conflicting. Low *TYMS* mRNA levels in CRC cells isolated from fresh specimens obtained after tumor resection were associated with in vitro resistance to 5-FU in those patients [109]. Moreover, low TYMS expression detected by immunohistochemistry was associated with a worse outcome in CRC patients treated with this drug [110]. However, other studies have reported that higher *TYMS* mRNA and protein expression was associated with poor response to 5-FU alone [111] and 5-FU-based regimens [112]. Besides, no correlation between immunohistochemical score of TYMS expression and the response to capecitabine of patients with advanced CRC was found; still, there was an association between the response and the predominant immunohistochemical staining pattern [113]. In the setting of advanced CRC, TYMS protein levels in the primary tumor tissue did not predict the response to 5-FU in a metastatic disease site [114].

Resistance to targeted therapy is linked to different processes: (i) activation of alternative tyrosine kinase receptors that bypass the main target, (ii) increased angiogenesis, (iii) constitutive activation of downstream mediators (revised in MOC-5), and iv) the presence of specific mutations in the target genes.

Cetuximab and panitumumab are monoclonal antibodies against the epidermal growth factor receptor (EGFR, gene *HER1*). They carry out high-affinity interactions with the EGFR extracellular domain, preventing the binding to its natural ligands. Both antibodies have been used in the treatment of wild-type *KRAS* metastatic CRC. However, some EGFR-expressing tumors are resistant to cetuximab. It has been proposed that the abundance of phosphorylated EGFR (pEGFR), which reflects better the receptor utilization by tumors, could be useful to predict the response of CRC to this drug; in fact, lower levels of pEGFR detected by immunohistochemistry were found in CRC patients with a more unsatisfactory response to cetuximab-based therapy [115]. Besides, a low copy number of *EGFR* has been associated with resistance to both cetuximab and panitumumab in patients with wild-type *KRAS* metastatic CRC [116]. Around 5% of metastatic CRC tumors are driven by amplification or mutation of epidermal growth factor 2 gene (*ERBB2*, also known as *HER2*). The HERACLES trials showed that ERBB2 inhibitors are an emerging treatment option in CRC [117]. It has been described that high ERBB2 expression levels detected by immunohistochemistry were associated with cetuximab resistance in metastatic CRC and that the presence of the R784G mutation, which lies within the protein kinase domain of the protein, correlated with lower survival rate [118].

Inhibition of angiogenesis is considered an effective strategy to treat metastatic CRC. Bevacizumab is a monoclonal antibody that targets vascular epidermal growth factor A (VEGF-A), preventing its binding with the VEGF receptor (VEGFR, *KDR* gene). Increased levels of VEGF-A in tumor or plasma were associated with poor prognosis, but the association with the response to bevacizumab has been controversial [119]. VEGF-A is overexpressed in hepatic metastasis of CRC after treatment with bevacizumab [120]. Besides, high VEGF-A serum levels have been correlated with resistance to bevacizumab [121]. Moreover, elevated plasma levels of soluble VEGFR-1 (*FLT1* gene) have been associated with a worse response in patients with CRC treated with bevacizumab [122].

Aflibercept is a recombinant fusion protein that binds VEGF-A, VEGF-B, and placental growth factor (PlGF), hindering their binding to VEGFR. By using a colorectal patient-derived xenograft model, in 39 out of 48 patient-derived tumors analyzed, aflibercept was more effective than bevacizumab [123]. A recent study confirmed that high VEGF-A and PlGF serum levels were associated with resistance to bevacizumab in patients with metastatic CRC, whereas aflibercept was active regardless of serum levels of these factors [121]. The increase in angiogenic factors in plasma, such as PlGF or VEGF-D, suggests that activation of other angiogenic pathways could play a role in the mechanism of resistance to VEGF-A blockade [124]. 

Ramucirumab is a monoclonal antibody that specifically targets VEGFR-2, blocking the binding of its ligands and inhibiting angiogenesis. Information regarding resistance to this drug is scarce. However, a case report study explained that the somatic missense mutation T771R in the *KDR* gene, encoding VEGFR-2, leads to self-activation of the receptor and thus stimulates angiogenesis. This contributed to acquired drug resistance in a patient with advanced CRC treated with ramucirumab-containing therapy [125].

Regorafenib, a multikinase inhibitor approved by the FDA as second-line therapy in CRC, interacts with tyrosine kinase receptors involved in angiogenesis (VEGFR 1-3, TIE-2), oncogenesis (c-KIT, RET), stromal signaling (PDGFR-β, FGFR1), and intracellular signaling (c-RAF/RAF-1, BRAF). The presence of the c.2881C > T mutation (R961W) in the *KDR* gene was associated with an exceptional response to regorafenib in a patient with advanced CRC highly resistant to 5-FU and bevacizumab [126]. Another case report described a patient suffering from CRC expressing wild-type BRAF, which was resistant to a regimen containing folinic acid, 5-FU, oxaliplatin, and bevacizumab, showed a long-term response to regorafenib [127].

## 5. DNA Repairing (MOC-4)

Several drugs used in the treatment of CRC can directly or indirectly induce severe DNA damage that triggers apoptosis. Effective DNA repair can remove drug-induced DNA alterations, allowing cancer cells to survive and proliferate. Therefore, chemoresistance may depend on the enhanced capacity of tumor cells to repair the DNA damage, whose underlying mechanisms have been classified as MOC-4 (Table 4).

Nucleotide excision repair (NER) is one of the primary mechanisms involved in repairing bulky DNA adducts, such as those produced by platinated drugs or alkylating agents. Among the essential proteins that participate in NER, ERCC1 (excision repair cross-complementing 1) has been identified as a candidate biomarker for predicting the efficacy of oxaliplatin in metastatic CRC. Patients with tumors expressing higher levels of ERCC1 and treated with oxaliplatin-based therapy had reduced OS, as well as a lower probability of 5-year disease-free survival (DFS) than those with lower ERCC1 expression [128]. The dysregulation of other ERCC family members has also been involved in CRC chemoresistance. Thus, elevated expression of ERCC6 in tumor tissue has been associated with poor OS and resistance to 5-FU chemotherapy [129].

Other components of the machinery responsible for NER are XPA and XPC (*Xeroderma pigmentosum* group A and group C, respectively) proteins, which are differentially expressed in CRC and may be involved in CRC chemoresistance. XPA interacts with a series of proteins to initiate the repairing process. XPA expression is significantly decreased in CRC tissues compared with adjacent nontumor tissues. Besides, high XPA expression shows a significant relationship with better survival of CRC patients, especially with rectal cancer [130]. On the contrary, XPC expression, also active at the early stage of DNA repair, acting as another DNA-damage-recognition protein, was markedly increased in CRC tissues compared with matched healthy controls. Moreover, it gradually increases along with CRC progression [131]. However, regarding the relationship between XPC expression and chemoresistance, conflicting results have been reported, as both enhanced resistance [131] and increased sensitivity [132] to cisplatin have been reported after XPC overexpression in CRC cells. Some clinical reports have shown that CRC patients with high XPC expression had longer survival, suggesting that XPC ameliorates prognosis by increasing the response to chemotherapy [132].

Several studies have shown that genetic variants of NER genes are relevant regarding clinical outcomes after oxaliplatin-based chemotherapy of CRC patients. Thus, rs11615 C > T (p.Asn118=) in *ERCC1* and rs13181 T > G (p.Lys751Gln) in *ERCC2* are SNPs with potential usefulness in the prognosis of CRC treated with oxaliplatin-based chemotherapy [133]. A study carried out in 126 patients with advanced CRC, treated with a first-line oxaliplatin/5-FU chemotherapeutic regimen, showed that rs11615 and rs13181 SNPs were significantly correlated with clinical response and OS [134]. Patients with the T/G haplotype of the *ERCC2* rs1799787 (c.1832-70C > T) and *ERCC1* rs10412761 (c.-22 + 1089T > G) SNPs had a 60% decrease in odds of response to preoperative capecitabine and oxaliplatin-based chemoradiotherapy in locally advanced rectal cancer [135]. *ERCC5* polymorphisms at the promoter region (c.-763A > G (rs2016073) and c.25G > A (rs751402)) may also be predictors of response to oxaliplatin-based chemotherapy in advanced CRC, as patients with the -763A/+25G haplotype had a higher risk of non-response to this pharmacological regimen [136]. 

The mismatch repair (MMR) system is a primary mechanism involved in the correction of point mutations, i.e., errors consisting of mismatched or wrongly matched nucleotides occurring during DNA replication. MMR is especially relevant in CRC, as the cause of approximately 15% of these tumors involves deficient mismatch repair (dMMR), which is characteristically accompanied by microsatellite instability (MSI) phenotype [137]. It has been extensively shown that dMMR is a predictive marker for the lack of efficacy of 5-FU-based adjuvant therapy in stages II and III of CRC. Patients with dMMR tumors seem less prone to benefit from 5-FU-based chemotherapy, while a better response was observed in patients with MMR-proficient tumors [138,139]. The dMMR phenotype is also predictive of resistance to oxaliplatin-based chemotherapy in metastatic CRC [140]. Although, in CRC, the presence of dMMR is associated with a weaker response to neoadjuvant chemotherapy (5-FU/oxaliplatin), these tumors are more sensitive to chemoradiation [141]. Moreover, in patients with metastatic CRC receiving irinotecan-based chemotherapy as the first-line regimen, a relationship between increased sensitivity to irinotecan and dMMR status has been reported [142]. Therefore, clinical findings appear to grant that CRC patient stratification based on MMR status may provide a more tailored approach for CRC therapy, and it should be considered in treatment decision making. 

Genetic variations affecting some of the proteins that integrate the MMR system, including MLH1 (MutL homolog 1), MLH3 (MutL homolog 3), MSH2 (MutS homolog 2), and MSH3 (MutS homolog 3), are frequent in CRC and have been associated with its degree of chemoresistance. In locally advanced rectal cancer patients receiving capecitabine-based neoadjuvant chemoradiotherapy, the intron variants rs175057 C > T (in *MLH3*) and rs13019654 G > T (in *MSH2*) can predict the response to treatment. Accordingly, these variants have been proposed as potential genetic markers in the personalized therapy of this cancer [143]. 

Base excision repair (BER) is a multi-step DNA repair pathway acting on damaged bases generated by alkylation, oxidation, or deamination. Together with MMR, BER plays a crucial role in the cellular responses to 5-FU treatment, recognizing and removing uracil and 5-FU from DNA. It has been shown that the efficacy of 5-FU in dMMR CRC cells is mostly dependent on BER, as these cancer cells have an increased requirement of the BER pathway for the efficient repair of 5-FU-induced DNA damage [144]. High expression of BER proteins is associated with more aggressive tumor features and poor outcomes in CRC patients [145]. However, in vitro overexpression of some BER proteins, such as MPG (N-methylpurine-DNA glycosylase), but not of others, such as XRCC1 (X-ray repair cross-complementing group 1), results in increased sensitivity of CRC cells to both 5-FU and the alkylating agent temozolomide, suggesting that BER modulation can alter the response to drug-induced DNA damage, making it a promising target for CRC therapy [145].

## 6. Balance between Pro-Apoptotic and Pro-Survival Factors (MOC-5)

A common feature in CRC cells is their ability to avoid apoptosis in response to anticancer drugs. This situation may be the consequence of a decrease in and/or defective function of pro-apoptotic mediators (MOC-5a) or an aberrant expression and/or function of anti-apoptotic proteins (MOC-5b) (Table 5).

### 6.1. Pro-Apoptotic Factors (MOC-5a)

The tumor-suppressor protein p53 (TP53 gene), which triggers cell arrest and apoptosis in response to DNA damage, presents polymorphisms and mutations that occur in up to half of CRC cases [146]. TP53 is the gene with the highest mutation rate in CRC [147]. The proportion of patients bearing TP53 alterations is higher in CRC than in other intestinal adenocarcinomas [148]. The results of in vitro assays have associated those mutations with a lower sensitivity of CRC cells to 5-FU [149] and weak response in patients with metastatic CRC treated with this drug [150]. 

Sensitivity to oxaliplatin may also be dependent on p53 activity. Cells with mutated TP53 showed reduced susceptibility to oxaliplatin [151]. Both in vitro and in vivo assays have indicated that the deletion of p53 upregulates miR-503-5p, inducing oxaliplatin resistance through apoptosis inhibition by reducing PUMA expression [152].

In association with other MOCs, some data indicate that the existence of mutated p53 in CRC correlates with MDR1 and GSTP overexpression [153]. Using adenovirus to induce wild-type p53 expression in CRC cells resulted in MDR1 downregulation and enhanced sensitivity to 5-FU [154]. 

The impact of mutations in the TP53 gene on the clinical response to chemotherapy in CRC patients remains under investigation because conflicting results have been reported. TP53 mutations were associated with 5-FU refractoriness in stage III CRC [155]. Besides, another study found that patients with impaired TP53 expression showed poor survival after FOLFOX treatment [156]. On the other hand, it has been suggested that TP53 mutations have no impact on the response to oxaliplatin- or irinotecan-based therapy in metastatic CRC [157].

Several p53 actions are mediated by p21, which participates in cell cycle arrest in response to DNA damage. It has been described that p21 downregulation through miR-520g confers resistance to 5-FU and oxaliplatin in CRC cells [158].

Alterations in apoptosis activation through the intrinsic pathway may also confer chemoresistance. In this sense, low expression and the presence of inactivating mutations affecting the pro-apoptotic gene BAX have been described in CRC patients. BAX expression has been proposed as a prognosis marker for 5-FU response [159,160]. Some strategies have been developed for patients with 5-FU-resistant tumors expressing low levels of BAX. For example, andrographolide, a natural diterpenoid, has been used to stimulate BAX expression and hence re-sensitize 5-FU-resistant CRC cells [159]. Elevated BAX expression levels in healthy intestinal tissue have been related to a higher apoptosis rate and a lower incidence of tumor development [161].

The abundance of pro-apoptotic proteins BAD and BID in CRC correlates with the response to 5-FU based therapies; hence, high expression of these proteins was associated with better response [162]. Furthermore, the experimental overexpression of FADD, an element of the extrinsic apoptosis pathway, increased the efficacy of 5-FU in CRC cells [163].

### 6.2. Survival Pathways (MOC-5b)

While the pro-apoptotic protein BAX is downregulated in CRC, BCL-2 (an anti-apoptotic element) is overexpressed [164], which is generally associated with drug resistance. In vitro studies have shown that BCL-2 inhibition (through miR-1915 overexpression) sensitized CRC cells to some anticancer drugs [165], such as 5-FU [166,167]. Inhibitors of BCL-2, BCL-w, or BCL-xL are under investigation to be used in CRC treatment along with conventional chemotherapy [168]. It has also been found that IL-17 promotes cell proliferation and inhibits apoptosis by enhancing the expression of p-AKT, mTOR, and BCL-2 and suppressing the expression of BAX, leading to cisplatin resistance [164].

The IAP (inhibitor of apoptosis protein) family acts by blocking caspase activity. The enhanced expression of IAPs (XIAP, cIAP1, cIAP2, and survivin) contributes to carcinogenesis and poor prognosis in CRC [169,170]. Downregulation of cIAP2 in CRC cells efficiently enhanced apoptosis through the activation of caspase 3/7 and hence increased the sensitivity of these cells to 5-FU [171]. 

MCL-1, another anti-apoptotic protein, may have a role in CRC chemoresistance, and its perinuclear staining has been associated with 5-FU therapy response [172].

The checkpoint kinases CHK1 and CHK2 are central control elements for cell cycle arrest, DNA damage checkpoint, DNA repair, and apoptosis activation. Elevated CHK1 expression predicted poor survival in CRC patients, especially under oxaliplatin treatment [173]. Besides, the inhibition of CHK1 might re-sensitize 5-FU-resistant CRC cells [174].

The NF-kB pathway is often constitutively activated in CRC [175], which has been associated with enhanced drug resistance. The inhibition of this pathway in CRC cells resulted in an increased cytotoxic effect of 5-FU [176,177] and gemcitabine [175]. Moreover, agents able to activate NF-κB signals, such as biglycan and CD133, enhance drug resistance through this pathway [178,179].

The Wnt/β-catenin pathway also plays a role in CRC chemoresistance, since in 5-FU-resistant CRC cells, higher expression of TCF4 and β-catenin has been found, which suggests a more active Wnt/β-catenin pathway and hence enhanced cell proliferation [188]. Inactivating mutations in the *APC* (adenomatous polyposis coli) gene also leads to constitutive activation of the Wnt/β-catenin pathway in CRC [189]. In fact, approximately 80% of CRC tumors harbor somatic inactivating mutations in the *APC* gene [190]. In CRC patients, *APC* mutations have been associated with worse responses to 5-FU [180]. Notably, the mutation rate of *APC* is markedly higher in CRC than in small intestine adenocarcinoma [148]. Nevertheless, some inactive variants of Wnt repressors RNF43 and ZNRF3 could lead to the activation of this pathway in other intestinal tumors [187].

Along with these pathways, Notch signaling is aberrantly activated in CRC [191], and its ligand Jagged1 also presents high expression in CRC [192]. This pathway controls the expression of cyclooxygenase-2 (COX-2), which is often upregulated in aggressive CRC and linked to a weaker response to 5-FU [185] and cisplatin due to the induction of MDR1 and MRP1 expression [186].

The mutation status of oncogenes RAS (KRAS or NRAS) and BRAF is a crucial factor determining the chemoresistance of CRC, where their alterations are associated with poor OS [193]. Patients with metastatic CRC whose tumors contain activating mutations in KRAS and NRAS present a lack of response to EGFR monoclonal antibody therapy [183,184]. Testing activating mutations in KRAS/NRAS remains the gold standard for the selection of therapeutic regimes with the EGFR targeted monoclonal antibodies cetuximab and panitumumab [194,195]. 

BRAF shows characteristic mutations in CRC. Thus, the canonical oncogenic p.V600E variant (c.1799T > A, rs113488022) was found in the vast majority of CRC patients, contrary to adenocarcinoma of the small intestine, where it represents only 10.3% of BRAF mutations [148]. BRAF p.V600E variant increases the growth and spread of cancer cells and, although, in some types of cancer, such as melanoma or non-small cell lung cancer, BRAF inhibitors have clinical activity in the V600E variant, these inhibitors alone have limited activity in BRAF V600E-mutated CRC [182].

Preclinical and clinical studies showed that the lack of efficacy of single-agent BRAF (encorafenib) or dual BRAF and MEK inhibition (encorafenib plus binimetinib) in BRAF V600E-mutated colorectal cancer is related to rapid EGFR-mediated adaptive feedback. This information led to the development of a combination of BRAF, MEK, and EGFR inhibition therapy. The BEACON phase 3 trial demonstrates that the combination of encorafenib and binimetinib with the EGFR inhibitor cetuximab resulted in significantly longer OS and a higher response rate than standard therapy in patients with metastatic CRC with the BRAF V600E mutation [196].

## 7. Adaptation to the Tumor Microenvironment (MOC-6)

Interactions between CRC cells and tumor stroma, with different immune and metabolic characteristics and the presence of pro-angiogenic and anti-angiogenic signals, can affect the response to pharmacological therapy (Table 6). The heterogeneity regarding tumor microenvironment has justified the classification of CRC cases into three subgroups based on their microenvironment’s characteristics: inflamed-stromal-dependent, inflamed-non-stromal-dependent, and non-inflamed or cold [197]. 

Hypoxia is a hallmark of most solid tumors as a result of increased oxygen consumption, due to the rapid proliferation of tumor cells together with insufficient and heterogeneous oxygen supply to different regions of the tumor mass. Hypoxia-inducible factor-1α (HIF-1α) plays a crucial role in the mechanisms activated in response to hypoxia, including those that induce anticancer drug resistance [198]. In CRC cells grown under hypoxic conditions either in monolayers or in 3D spheroids, HIF-1α induced MDR1 expression and, consequently, resistance to several drugs. The high expression of HIF1α and MDR1 detected by immunohistochemistry has been associated with a lower response to 5-FU in patients with advanced CRC [199]. Moreover, in vitro and in vivo experiments with CRC cells demonstrated that the antitumor effect of bevacizumab was dependent on sensitivity to hypoxia-induced apoptosis [200].

Cancer-associated fibroblasts (CAFs) are essential components of CRC stroma that contribute to cancer cell proliferation but also drug resistance through changes induced by their released cytokines. Experiments with in vitro and in vivo models of patient-derived CRC revealed that HIF-1α and CAFs-secreted TGF-β synergistically induced the expression of the hedgehog transcription factor GLI2 (glioma-associated oncogene homolog 2), leading to 5-FU/oxaliplatin resistance [201]. Moreover, using cells isolated from primary CRC specimens, it was observed that the number of CAFs was increased after treatment with 5-FU/oxaliplatin and that cancer stem cells (CSCs) were protected from chemotherapy-induced growth inhibition by IL-17A secretion [202].

Different types of immune cells interact with cancer cells and other components of the tumor stroma through cytokine production, altering tumor growth and its response to drug therapy. It is worth noting that a subgroup of CRC is generated within the context of inflammatory bowel disease— in particular, ulcerative colitis. Tumor-associated macrophages (TAMs) are one of the most abundant types of immune cells affecting tumor progression by the production of pro- and anti-inflammatory cytokines. There are controversial results regarding the effect of TAMs on chemoresistance. One study found that a low abundance of these cells was associated with worse PFS in CRC patients receiving 5-FU adjuvant therapy [203]. In contrast, other authors have reported higher macrophage infiltration in CRC specimens collected from tumors resistant to 5-FU and oxaliplatin, which was associated with lower survival [204]. In vitro experiments revealed that IL-6 secretion by TAMs induced chemoresistance by activating the IL-6R/STAT3/miR-204-5p pathway in CRC cells [204]. Whether this effect depends on the presence of macrophages with M1 or M2 phenotype remains to be elucidated. The impact of subtypes of tumor-infiltrating lymphocytes (TILs) has also been investigated. Low tumor infiltration by regulatory T cells (Treg) and T lymphocytes expressing CCR7 (chemokine receptor 7), considered indicators of the local antitumor immune response, was associated with lower OS and PFS in CRC patients treated with FOLFOX or irinotecan [205].

Due to the anatomical location of CRC, the gut microbiota also contributes to the tumor microenvironment, having an impact on CRC initiation and progression by modulating intestinal inflammation, signaling pathways, and local immune response, which can affect the response to chemotherapy and immunotherapy [206]. A higher abundance of *Fusobacterium nucleatum* was associated with recurrence after treatment of CRC patients with oxaliplatin and capecitabine; the authors of that study demonstrated that the resistance was mediated by selective target loss of miR-18a* and miR-4802 and activation of the autophagy pathway [207]. 

Using CRC models in mice, resistance to gemcitabine was shown to be induced by its inactivation by the enzyme cytidine deaminase, present in gammaproteobacteria [208]. Altered microbiota impaired the response of several subcutaneous tumors in mice, including CRC, to platinum derivatives and CpG-oligonucleotide immunotherapy. This situation was explained by a deficient production of reactive oxygen species (ROS), cytotoxicity after chemotherapy, lower cytokine production, and tumor necrosis by tumor-infiltrating myeloid-derived cells [210].

Metabolic reprogramming is a main feature of cancer cells which permits them to enhance aerobic glycolysis over full oxidation of glucose in their mitochondria (Warburg effect), even with a normal mitochondrial function. In CRC cells resistant to oxaliplatin and 5-FU, high intracellular ATP levels have been associated with chemoresistance. Indeed, ATP depletion was sufficient to sensitize cross-resistant cells to multiple chemotherapeutic agents [211].

Exosomes are extracellular nanovesicles containing diverse biomolecules, such as proteins, lipids, and nucleic acids. They are involved in cell–cell communication with pleiotropic functions. It has been proposed that, by interacting with the tumor microenvironment, exosomes can participate in tumor progression, immune escape, angiogenesis, and drug resistance [212]. The lncRNA UCA1 (urothelial carcinoma-associated 1) was found overexpressed in cetuximab-resistant CRC cells, whose released exosomes could induce drug resistance in sensitive cells. Moreover, high levels of UCA1 in exosomes were associated with a lower response to cetuximab therapy in CRC patients [209]. Tumor cells are not the only ones that can modify the anticancer drug resistance of neighboring cells through exosomes; in fact, CAF-derived exosomes enhanced CSC resistance to 5-FU or oxaliplatin [213].

The extracellular matrix (ECM) surrounding CRC cells can also affect resistance to anticancer drugs. Higher resistance to vemurafenib was observed when CRC cells were cultured on conventional tissue culture plastic plates compared with those grown in 3D collagen I gels [214]. In addition, 3D in vitro models using type I collagen showed that a change in tumor morphology, from clonal cysts to spiky masses, reduced the response to cetuximab without affecting EGFR expression, through activation of the MET/RON survival pathways [215]. However, the contribution of ECM to drug resistance in clinical practice remains to be elucidated. 

## 8. Phenotype Transition (MOC-7)

CSCs contribute to CRC chemoresistance as they can self-renew and differentiate into heterogeneous lineages of cancer cells in response to pharmacological treatment [216]. Their ability to undergo cell cycle arrest and remain in a quiescent state increases their chance of becoming resistant to chemotherapy. Conventional drugs can suppress the bulk of proliferating tumor cells, but a group of CSCs can often survive and promote cancer relapse. CSCs can originate from adult stem cells by accumulating genetic or epigenetic abnormalities, which neutralize the limitations in the proliferation of healthy cells, or from differentiated cells that have acquired stem cell-like characteristics during dedifferentiation. Moreover, the induction of epithelial-to-mesenchymal transition (EMT) confers upon tumor cells some typical traits of CSCs. EMT is a complex process that in most cases is accompanied by a dysregulation of survival pathways, upregulation of stemness markers, and gain-of-function mutations in tumor suppressor genes, which altogether contribute to the association of EMT with the emergence of chemoresistance [217]. However, using CRC-derived cell lines, it has been demonstrated that in early stages of the EMT process, such as only to the loss of E-cadherin, there is an increased sensitivity to 5-FU, irinotecan and oxaliplatin [218] (Table 7).

CD133 and CD44 are two of the most documented markers of CSCs and EMT cells related to the development of chemoresistance in CRC [219,220]. Other relevant stemness markers that have been associated with a worse response of CRC patients to platinum- and pyrimidine-based regimens are CD262 [221] and LGR5 [222]. In cells with upregulation of cell adhesion markers, there is also an overactivation of survival signaling pathways [223], resulting in increased expression of anti-apoptotic factors such as survivin (MOC-5b) [219]. The dependence of tumor cells on the high activity of some survival pathways, such as that activated by VEGF, can be exploited as a pharmacological strategy of targeted therapy. In this sense, patients with high CD133 expression, which induces upregulation of VEGF and its receptors, respond better to bevacizumab [224]. It has been suggested that CD47 expression in CRC allows cancer cells to escape the antitumor attack carried out by the innate immune system. In fact, recurrent tumors that appear after treatment with nivolumab, an antibody against the programmed cell death ligand 1 (PD-L1), undergo phenotypic transition, which is associated with higher expression of CD47 and CD44 [225].

Aldehyde dehydrogenase (ALDH), a cytosolic enzyme that protects cells from the potentially toxic effects of ROS, is highly expressed in CSCs, where it plays an essential role in their chemoresistance [226]. In CRC patients treated with adjuvant 5-FU therapy, high levels of claudin-2, associated with a population of CSCs with high expression of ALDH, correlated with lower recurrence-free survival (RFS) [227].

The high expression of TWIST1 is one of the hallmarks of EMT. As this gene is involved in the increased proliferation and chemoresistance of CRC cells, it has been proposed as a potential biomarker of prognosis for patients with CRC [228]. 

*OCT4*, *SOX2*, and *NANOG* genes collaborate to control the expression of other genes related to pluripotency. Their high expression in CRC has been associated with a weaker response of patients to conventional chemotherapy [243]. *SNAI1* and *ZEB2* play a similar role, as their overexpression promotes CRC recurrence by inducing EMT [242,244]. Some translation initiation factors that control EMT, such as eIF4E and eIF5A2, contribute to tumor malignancy by enhancing chemoresistance [240,241]. 

Dysregulation of some signaling pathways can trigger EMT as well as the development of CSCs in CRC. An aberrant Wnt/β-catenin pathway can be one of those circumstances. The loss of nuclear expression of Dickkopf-1, an extracellular inhibitor of this pathway, has been associated with a decrease in OS in CRC patients treated with 5-FU or FOLFOX/FOLFIRI regimens [239]. Moreover, both in vitro and in vivo studies have suggested that increased USP22 (ubiquitin-specific peptidase 22) expression in CSCs derived from CRC is responsible for resistance to 5-FU due to the induction of the Wnt/β-catenin pathway [245].

The induction of EMT due to the overactivation of the Notch signaling pathway is involved in the development of chemoresistance in CRC. The overexpression of HES1, one of the target genes of this pathway, has been found in CRC patients treated with adjuvant 5-FU-based therapy, who had lower OS and PFS [237]. 

Besides, high expression of Jagged-1 ligand and APEX1 has been associated with a worse response of CRC patients to 5-FU, oxaliplatin, and irinotecan [238]. The upregulation of upstream elements of the Notch pathway, such as ADAM17 and ADAM10, could be one of the mechanisms responsible for the high activity of this pathway. Pharmacological inhibition of ADAM17 in vitro and in vivo has demonstrated its ability to sensitize CRC cells to 5-FU and irinotecan, in addition to reversing the EMT phenotype [246]. Downregulation of NUMBL, an inhibitor of Notch, in CRC cells induced activation of this pathway and hence also triggered EMT, acquisition of stem-cell-like properties in tumor cells, as well as increased chemoresistance [247].

The hedgehog signaling pathway is a potential target for reverting EMT and sensitizing CRC to anticancer drugs. In organoids derived from CRC, inhibitors of the hedgehog pathway enhance sensitivity to 5-FU, irinotecan, and oxaliplatin [248]. 

Activation of the EGFR pathway also contributes to the chemoresistance of CRC by inducing EMT and enriching the CSC population [249]. Similarly, the reduced expression of this pathway’s inhibitors, such as VPS33B, has been associated with poorer prognosis in patients and increased resistance to oxaliplatin in vitro and in vivo [236].

EMT is also regulated in CRCs by a variety of ncRNAs. In some cases, the loss of miRNAs has been associated with worse patient response to pharmacological treatment. Some examples include miR-128-3p [230], miR-200a, miR-200c, miR-429 [232], and miR-324-5p [229]. In other cases, the upregulation of several ncRNAs, such as miR-27b [231], miR-92a-3p [234], miR-148 [231], miR-205 [233], miR-373 [233], and the lncRNA MALAT1, has been associated with a more aggressive phenotype and poorer patient response [235]. Levels of miRNAs involved in the induction of a phenotypical transition in CRC cells have also been proposed as plasma markers to predict the lack of response to chemotherapy in CRC patients [231,234].

## 9. Conclusions and Perspectives

The information analyzed in the present review article permits us to reach several interesting conclusions. In the first place, it is evident the existence of a high degree of complexity regarding MOCs and how they interact and synergize with each other, rendering CRC cells strongly resistant to the available pharmacological armamentarium. Moreover, it is currently well-known that tumors are neither homogeneous nor static. Therefore, both characteristics, i.e., cellular heterogeneity and dynamic changes in tumor cell phenotype, must be considered for a better understanding of the lack of CRC response to pharmacological treatment. As new strategies to overcome this refractoriness are required, to advance toward a better understanding of the problem is essential in order to identify cancer cell weaknesses. This will permit us to develop sensitizing tools which may include (i) new agents to be co-administered with the active drug; (ii) pharmacological approaches, such as drug encapsulation (e.g., into labeled liposomes or exosomes); (iii) gene therapy interventions aimed at restoring the impaired function of some proteins (e.g., uptake transporters and tumor suppressors) or abolishing that of others (e.g., export pumps and oncogenes).

## Figures and Tables

**Table 1:** Mechanisms of chemoresistance type 1 (MOC-1) in colorectal cancer.

Protein	Change	Drugs Affected	Consequences	References
Uptake Transporters (MOC-1a)				
OATP1B1	GV (OATP1B1*15 haplotype)	Irinotecan, Methotrexate	Lower response in vitro and in patients	[11,15,16,18,19]
OATP1B3	GV (Cancer-type)	Irinotecan	Reduced PFS	[21,22,23]
OATP1A2	Downregulation	Imatinib, Methotrexate	Reduced drug uptake	[16,27]
OCT1	Downregulation	Imatinib, Doxorubicin	Lower sensitivity in vitro; lower clinical response	[32,33,34,35]
OCT3	Impaired expression	Irinotecan, Imatinib, Cisplatin, 5-FU, FOLFOX	Lower clinical response	[38,39]
OCTN2	GV (rs2631367, rs2631372)	Imatinib, Etoposide	Lower sensitivity in vitro	[38,42,43,44]
CTR1	Downregulation	Cisplatin	Lower sensitivity in vitro	[60]
Efflux transporters (MOC-1b)				
MDR1	Upregulation	Doxorubicin, Etoposide, Irinotecan	Lower sensitivity in vitro	[47]
MRP1	Upregulation	Doxorubicin, Etoposide, 5-FU, Oxaliplatin	Lower sensitivity in vitro	[61,62]
MRP2	Upregulation	Cisplatin	Lower sensitivity in vitro	[56]
MRP3	Upregulation	Doxorubicin, Etoposide	Lower sensitivity in vitro	[63]
MRP4	GV (rs3742106)	5-FU, Capecitabine	Lower clinical response	[64]
MRP5	Upregulation	5-FU, Methotrexate	Lower sensitivity in vitro	[65]
BCRP	GV (rs2231137, rs2231142)	Irinotecan	Lower sensitivity in vitro; Lower clinical response	[66,67]
ATP7B	Upregulation	Oxaliplatin	Poor clinical outcome	[68]
ABCA9	GV	Oxaliplatin	Reduced OS and response	[69]
LRP	Upregulation	Doxorubicin, Etoposide	Lower sensitivity in vitro	[70,71]

5-FU: 5-fluorouracil; FOLFOX: leucovorin (folinic acid), 5-FU, and oxaliplatin regimen; GV: genetic variant; OS: overall survival; PFS: progression-free survival.

**Table 2:** Mechanisms of chemoresistance type 2 (MOC-2) in colorectal cancer.

Protein	Change	Drugs Affected	Consequences	References
CYP3A5, CYP3A4	Upregulation	Irinotecan (SN-38)	Enhanced drug inactivation	[79,80]
CYP1A2, CYP2A6	Upregulation	5-FU	Enhanced drug inactivation	[81]
CES2	Downregulation	Irinotecan	Reduced drug activation	[82,84]
DPYP	Upregulation	5-FU	Reduced clinical response	[87,88]
TYMP	Downregulation	5-FU	Reduced drug activation	[87]
γ-GCS	Upregulation	Cisplatin, Doxorubicin	Enhanced drug inactivation	[92,93]
GSTA1	Upregulation	Irinotecan (SN-38)	Enhanced drug inactivation	[94]
GSTO1	Upregulation	Cisplatin	Enhanced drug inactivation	[95]
GSTP1	Upregulation	Anthracyclines	Enhanced drug inactivation	[92]
UGTs	Upregulation	Irinotecan (SN-38)	Enhanced drug inactivation	[96,97]
MT	Upregulation	Cisplatin	Reduced sensitivity in vitro and poor clinical prognosis *	[98,99]

5-FU: 5-fluorouracil; GV: gene variant; *: contradictory data.

**Table 3:** Mechanisms of chemoresistance type 3 (MOC-3) in colorectal cancer.

Protein	Change	Drugs Affected	Consequences	References
EGFR	Low gene copy number	Cetuximab Panitumumab	Reduced response in patients with wild-type KRAS	[116]
pEGFR	Low levels	Cetuximab	Reduced clinical response	[115]
ERBB2	Upregulation and R784G mutation	Cetuximab	Reduced clinical response	[118]
PlGF	High serum levels	Bevacizumab	Reduced clinical response	[121]
TYMS	Downregulation	5-FU	Worse outcome *	[109,110]
VEGF-A	High serum levels	Bevacizumab	Reduced clinical response	[121]
VEGFR-1	High serum levels	Bevacizumab	Reduced clinical response	[122]
VEGFR-2	T771R mutation	Ramucirumab	Reduced clinical response	[125]

5-FU: 5-fluorouracil; *: contradictory data.

**Table 4:** Mechanisms of chemoresistance type 4 (MOC-4) in colorectal cancer.

Protein	Change	Drug Affected	Consequences	References
Nucleotide Excision Repair (NER)				
ERCC1	High expression	Oxaliplatin	Reduced efficacy	[128]
ERCC1	GV (rs11615, rs10412761)	Oxaliplatin, 5-FU, Capecitabine	Reduced efficacy	[133,134,135]
ERCC2	GV (rs13181, rs1799787)	Oxaliplatin, 5-FU, Capecitabine	Reduced efficacy	[133,134,135]
ERCC6	High expression	5-FU	Reduced efficacy	[129]
XPC	High expression	Cisplatin	Drug resistance *	[131,132]
Mismatch Repair (MMR)				
Several	Defective MMR	5-FU, Oxaliplatin	Reduced efficacy	[138,139,140]

5-FU: 5-fluorouracil; GV: genetic variants; *: controversial data.

**Table 5:** Mechanisms of chemoresistance type 5 (MOC-5) in colorectal cancer.

Protein	Change	Drugs Affected	Consequences	References
Pro-Apoptotic Factors (MOC-5a)				
BAD	Downregulation	5-FU	Apoptosis inhibition	[162]
BAX	Downregulation and inactivating mutations	5-FU	Apoptosis inhibition	[159,160]
BID	Downregulation	5-FU	Apoptosis inhibition	[162]
FADD	Downregulation	5-FU	Apoptosis inhibition	[163]
miR-520g	Upregulation	5-FU, Oxaliplatin	No cell cycle arrest; apoptosis inhibition; p21 downregulation	[158]
p53	Inactivating mutations	5-FU, FOLFOX	No cell cycle arrest; apoptosis inhibition	[149,150,155,156]
		Oxaliplatin	miR-503-5p upregulation; PUMA downregulation; apoptosis inhibition	[151,152]
		5-FU	Associated with enhanced MDR1 and GSTP expression	[153,154]
Survival Pathways (MOC-5b)				
APC	Inactivating mutations	5-FU	Stimulation of Wnt/β-catenin	[180]
BCL-2	Upregulation	5-FU	Apoptosis inhibition	[166,167]
Biglycan	Upregulation	5-FU	Increased activity of the NFκB pathway	[178]
BRAF	Inactivating mutations	Vemurafenib, Dabrafenib, Encorafenib	Increased proliferation	[181,182]
CD133	Upregulation	Doxorubicin	Increased activity of the NFκB pathway; MDR1 upregulation	[179]
CHK1	Upregulation	5-FU, Oxaliplatin	No cell cycle arrest; apoptosis inhibition	[173,174]
IAP2	Modulation of caspase 3/7 activity	5-FU	Apoptosis inhibition	[171]
IL-17	Upregulation of p-AKT, mTOR and BCL-2; Suppression of BAX	Cisplatin	Apoptosis inhibition	[164]
KRAS	Activating mutations	Cetuximab, Panitumumab, others	Increased proliferation	[148,183,184]
MCL-1	Perinuclear expression	5-FU	No cell cycle arrest; apoptosis inhibition	[172]
NFκB	Increased activity	5-FU, Gemcitabine	Upregulation of anti-apoptotic factors	[175,176,177]
Notch	Increased activity	5-FU, Cisplatin	Upregulation of COX2; MDR1 and MRP1 upregulation	[185,186]
RNF43	Inactivating mutations	Dacomitinib	Stimulation of Wnt/β-catenin	[187]
Wnt/β-catenin	Increased activity	5-FU	Stimulation of cell proliferation	[188]
ZNRF3	Inactivating mutations	Dacomitinib	Stimulation of Wnt/β-catenin	[187]

5-FU: 5-fluorouracil; FOLFOX: oxaliplatin/leucovorin (folinic acid)/5-FU regimen.

**Table 6:** Mechanisms of resistance type 6 (MOC-6) in colorectal cancer.

Factor	Change	Drugs Affected	Consequences	Reference
HIF-1α	Upregulation	5-FU	MDR1 upregulation; lower response to treatment	[199]
	Upregulation	Bevacizumab	Lower apoptosis in resistant cells in vitro	[200]
HIF-1α, TGF-β	High expression	5-FU, Oxaliplatin	Increased GLI2 expression; lower drug effect in vitro	[201]
IL-17A	Increased production	5-FU, Oxaliplatin	Reduced drug effect on CSCs	[202]
Gut microbiota	*Fusobacterium nucleatum*	Oxaliplatin, Capecitabine	Lower response to treatment	[207]
	Gammaproteobacteria	Gemcitabine	Drug inactivation; reduced efficacy in vivo	[208]
UCA1	Upregulation	Cetuximab	Reduced drug efficacy in vitro and in patients	[209]

5-FU: 5-fluorouracil; CSC: cancer stem cells.

**Table 7:** Mechanisms of chemoresistance type 7 (MOC-7) in colorectal cancer.

Factor	Change	Drugs Affected	Consequences	References
Cell Adhesion Proteins				
CD133	Downregulation	Bevacizumab	Increased DPR	[224]
CD133	Upregulation	5-FU	Reduced sensitivity in vitro	[219]
CD262	Upregulation	5-FU, Cisplatin	Reduced sensitivity in vitro	[221]
CD44	Upregulation	5-FU, Oxaliplatin	Reduced sensitivity in vitro and in vivo	[220]
CD44, CD47	Upregulation	Nivolumab	Reduced DFS	[225]
E-cadherin	Downregulation	5-FU, Irinotecan, Oxaliplatin	Higher sensitivity in vitro	[217]
LGR5	Upregulation	5-FU, Capecitabine, Oxaliplatin	Reduced DFS and OS	[222]
Enzymes				
ALDH	Upregulation	5-FU	Reduced RFS	[227]
Non-Coding RNAs				
miR-324-5p	Downregulation	5-FU, Oxaliplatin	Reduced clinical response	[229]
miR-128-3p	Downregulation	Oxaliplatin	Reduced PFS	[230]
miR-148a, miR-27b	Upregulation	5-FU, Oxaliplatin	Reduced PFS	[231]
miR-200a, miR-200c, miR-429	Downregulation	Adjuvant chemotherapy	Reduced OS	[232]
miR-205, miR-373	Upregulation	5-FU, Oxaliplatin	Increased cancer progression	[233]
miR-92a-3p	Upregulation	5-FU, Oxaliplatin	Reduced clinical response	[234]
MALAT1	Upregulation	5-FU, Oxaliplatin	Reduced OS and PFS	[235]
Survival Pathways				
EGFR	Overactivation	Oxaliplatin	Reduced sensitivity in vitro and in vivo	[236]
Notch	Overactivation	5-FU, Oxaliplatin, Irinotecan	Reduced DFS and OS	[237,238]
Wnt/β-catenin	Overactivation	5-FU, Oxaliplatin, Irinotecan	Reduced OS	[239]
Transcription Factors				
eIF4E	Upregulation	5-FU	Reduced sensitivity in vitro	[240]
eIF5A2	Upregulation	Doxorubicin	Reduced sensitivity in vitro	[241]
SNAI1	Upregulation	5-FU, Paclitaxel	Reduced sensitivity in vitro and in vivo	[242]
SOX2, OCT4, NANOG	Upregulation	5-FU, Oxaliplatin	Reduced OS and RFS	[243]
TWIST1	Upregulation	5-FU, Oxaliplatin	Reduced OS	[228]
ZEB2	Upregulation	5-FU, Oxaliplatin	Reduced RFS	[244]

5-FU: 5-fluorouracil; DFS: disease-free survival; DPR: disease progression rate; OS: overall survival; PFS: progression-free survival; RFS: recurrence-free survival.

## Abbreviations

5-FU, 5-fluorouracil; ABC, ATP-binding cassette; ALDH, aldehyde dehydrogenase; APC, adenomatous polyposis coli; ATP7, ATPase copper transporting; BCL, B-cell lymphoma 2 family of apoptosis regulator proteins, BCRP, breast cancer resistance protein; BER, base excision repair; CAF, cancer-associated fibroblast; CES, carboxylesterase 2; CHK, checkpoint kinases; COX-2, cyclooxygenase-2; CRC, colorectal cancer; CSC, cancer stem cell; CTR1, copper transport protein; CYP, cytochrome; DFS, disease-free survival; DPD, dihydropyrimidine dehydrogenase; dMMR, deficient mismatch repair; DPR, disease progression rate; ECM, extracellular matrix; EGFR, epidermal growth factor receptor; EMT, epithelial–mesenchymal transition; ERCC1/2, excision repair cross-complementation 1/2; FOLFIRI, folinic acid/5-FU/irinotecan regimen; FOLFOX, folinic acid/5-FU/oxaliplatin regimen; γ-GCS, γ-glutamylcysteine synthetatase; GST, glutathione-S-transferase; HER1/2, human epidermal growth factor receptor 1/2; HIF, hypoxia-inducible transcription factor; IAP, inhibitor of apoptosis protein; IARC, International Agency for Research on Cancer; IL, interleukin; lncRNA, long non-coding RNA; LRP, lung resistance-related protein; MDR, multidrug resistance; MLH1, MutL homolog 1; MLH3, MutL homolog 3; MPG, N-methylPurine-DNA glycosylase; MSH2, MutS homolog 2; MSH3, MutS homolog 3; MMR, mismatch repair system; MOC, mechanism of chemoresistance; MPG, N-methylpurine-DNA glycosylase; MRP, multidrug resistance protein; MSI, microsatellite instability; MT, metallothionein; MTHFR, methylenetetrahydrofolate reductase; NER, nucleotide excision repair; NSAID, nonsteroidal anti-inflammatory drug; OATP, organic anion transporting polypeptide; OCT, organic cation transporter; OS, overall survival; pEGFR, phosphorylated EGFR; PFS, progression-free survival; PlGF, placental growth factor; RFS, recurrence-free survival; ROS, reactive oxygen species; SLC, solute carrier; SNP, single nucleotide polymorphism; TAM, tumor-associated macrophage; TILs, tumor-infiltrating lymphocytes; TKI, tyrosine kinase inhibitor; TYMP, thymidine phosphorylase; TP53, tumor protein 53; TS, thymidylate synthase; TTP, time-to-progression; UCA1, urothelial carcinoma-associated 1; UGT, UDP-glucuronosyltransferases; USP22, ubiquitin-specific peptidase 22; VEGF, vascular endothelial growth factor; VEGFR-2, vascular endothelial growth factor receptor 2; WHO, World Health Organization; Wnt, wingless integration site; XELOX, oxaliplatin/capecitabin; XPA, xeroderma pigmentosum group A; XPC, xeroderma pigmentosum group C; XRCC1, X-ray repair cross complementing 1.
